# Efficacy, Safety, and Pharmacokinetics of a New 10 % Liquid Intravenous Immunoglobulin Containing High Titer Neutralizing Antibody to RSV and Other Respiratory Viruses in Subjects with Primary Immunodeficiency Disease

**DOI:** 10.1007/s10875-016-0308-z

**Published:** 2016-06-20

**Authors:** Richard L. Wasserman, William Lumry, James Harris, Robyn Levy, Mark Stein, Lisa Forbes, Charlotte Cunningham-Rundles, Isaac Melamed, Ai Lan Kobayashi, Wei Du, Roger Kobayashi

**Affiliations:** 1 Allergy Partners of North Texas Research, 7777 Forest Lane, Building B, Suite 332, Dallas, TX 75230 USA; 2 Allergy and Asthma Specialists, 10100 North Central Expressway, Suite 100, Dallas, TX 75231 USA; 3 The South Bend Clinic, LLP, 211 North Eddy Street, South Bend, IN 46617 USA; 4 Family Allergy & Asthma Center, PC, 5555 Peachtree Dunwoody Road NE, Atlanta, GA 30342 USA; 5 Allergy Associates of Palm Beaches, 840 US Highway 1, Suite 250, North Palm Beach, FL 33408 USA; 6 Baylor College of Medicine, Feigin Center, 1102 Bates Street, Suite 330, Houston, TX 77030 USA; 7 Icahn School of Medicine at Mount Sinai, 1425 Madison Avenue, New York, NY 10029 USA; 8 Immunoe International Research Centers, 6801 South Yosemite Street, Centennial, CO 80112 USA; 9 Midlands Pediatrics, P.C., 401 East Goldcoast Road, Suite 325, Papillion, NE 68046 USA; 10 Clinical Statistics Consulting, 110 Chatsworth Court, Blue Bell, PA 19422 USA; 11 UCLA, School of Medicine, 10833 Le Conte Avenue, Los Angeles, CA 90095 USA

**Keywords:** Intravenous immunoglobulin (IVIG), primary immunodeficiency (PIDD), clinical trial, safety, efficacy, pharmacokinetics

## Abstract

**Purpose:**

Immune globulins for IgG supplementation have been produced for over 35 years with essentially no differentiating features regarding their specific antibody composition. Furthermore, the compositions of plasma donor pools used for IG manufacturing are not standardized. While all immune globulin products meet the specifications set by the US FDA for antibodies to pathogens like measles and polio, they have variable levels of antibodies to other important viruses and infectious pathogens, particularly respiratory syncytial virus (RSV).

**Methods:**

An IVIG was developed that satisfies the requirements for treating patients with primary immune deficiency disease (PIDD) and also has standardized elevated levels of RSV neutralizing antibodies (RI-002). Plasma donors who have naturally occurring high circulating levels of neutralizing anti-RSV antibody were selected as the source for manufacturing IVIG to treat patients with PIDD to prevent serious bacterial infections. While the introduction of the monoclonal antibody Palivizumab has had a dramatic impact in diminishing the burden of RSV disease in the pediatric population, it does not meet the standards for replacing the deficient immune compartments of patients with PIDD.

**Results:**

Fifty-nine patients with PIDD at 9 different sites across the US were enrolled in this study and received regular infusions of RI-002 over the course of 1 year.

**Conclusions:**

There were zero serious bacterial infections, thus meeting the primary endpoint for this trial. The secondary endpoints including days missed from work due to infection, unscheduled visits to the physician, and days of hospitalization due to infection compared favorably to published reports of other IVIG products.

## Introduction

Immunoglobulin (IG) supplementation has been the standard treatment for patients with disorders of antibody production due to B cell or combined B and T cell abnormalities (PIDD) [1–5]. Regularly scheduled infusions of IG replace or supplement antibodies that decrease the risk of the serious bacterial and viral infections experienced by PIDD patients [6–8]. Although all available preparations of immune globulin provide sufficient antibodies to significantly decrease the frequency of infections in immunodeficient patients, infections continue to occur [2, 6]. Previous studies have suggested that some infections in antibody-deficient PIDD patients occur when the titer of protective antibody is inadequate [9–12]. While many of these reports have focused on bacterial pathogens, additional evidence supports the proposition that high titer anti-viral antibody preparations may provide advantages over conventional IG [13, 14].

Studies of an early RSV-IVIG, RespiGam®, in premature infants demonstrated the ability of this polyclonal anti-RSV hyperimmune globulin to reduce not only respiratory syncytial virus (RSV) infections but also other viral respiratory infections as well as otitis media [15, 16]. When this product was available, the American Academy of Pediatrics (AAP) stated that for children with severe immunodeficiencies receiving IG therapy during the winter months, physicians could consider the addition and/or substitution of the high titer RSV-IVIG for standard IG supplementation [17]. These data suggested that a polyclonal anti-RSV hyperimmune globulin would confer enhanced protection from infection. Despite the successful use of this RSV-IVIG in premature infants, it was voluntarily withdrawn after the introduction of a monoclonal anti-RSV (Synagis®) antibody that could be administered intramuscularly rather than intravenously as was required for the polyclonal antibody [14]. Consequently, a new IG formulation that meets all the standard criteria for treatment of PIDD (RI-002) was developed using plasma collected from individuals tested to have high titer anti-RSV antibodies [18].

The present study was a prospective, open-label, non-randomized multicenter phase 3 study in the USA to evaluate the efficacy and safety of RI-002 in patients with PIDD. The primary objective was to evaluate the annualized acute serious bacterial infection rate. Secondary objectives including evaluation of missed days of work due to infection, unscheduled visits to the physician, days hospitalized due to infection as well as the safety and tolerability of RI-002 were also studied. Pharmacokinetic studies measured not only concentrations of total immune globulin, but also measured the concentrations of antibodies to RSV, cytomegalovirus (CMV), tetanus toxoid, *Haemophilus influenzae* type b (Hib), measles, and 13 serotypes of *Streptococcus pneumoniae*.

## Methods

### Study Product

The investigational new drug used in this study (RI-002) was provided by ADMA Biologics (Ramsey, NJ).

RI-002 was manufactured from source plasma, obtained from approved donors, and screened for blood borne pathogens by binding assays and nucleic acid testing, which was purchased and obtained from domestic plasma donation centers licensed by US FDA and certified by Plasma Protein Therapeutic Association (PPTA). The plasma pool for each lot of RI-002 included sufficient plasma from donors with high neutralizing titers to RSV to meet a standardized value.

The manufacturing method included Cohn-Oncley fractionation followed by ion exchange chromatography followed by other downstream purification steps. The final product contains 100 ± 10 mg/mL protein. At least 96 % of the protein is human IgG with a monomer plus dimer content greater than 95 %. The distribution of IgG subclasses in the final product is similar to the distribution in the starting plasma.

The potency of the product meets 21 CFR 640.104 criteria for the required levels of antibody to measles, polio, and diphtheria in addition to having standardized elevated neutralizing titers to RSV. The RSV neutralizing titers for all lots used in the study were similar and have been found to be comparable to the former RespiGam® product.

### Study Design

This was an open-label, phase III study to evaluate the efficacy, safety, and pharmacokinetics of subjects with documented PIDD (clinicaltrials.gov NCT01814800). Intravenous infusions were given at the study centers at doses of 300–800 mg/kg every 3 or 4 weeks for approximately 1 year. Dosing interval was determined by each subject’s pre-study infusion schedule. Infusion rates began at 0.5 mg/kg/min and were incrementally increased every 15 min until reaching a maximum of 8 mg/kg/min. Doses of RI-002 were adjusted during the study to maintain minimum trough IgG concentrations >500 mg/dL or at the investigator’s discretion.

A subset of subjects participated in the PK portion of the study. PK data was analyzed for total IgG concentrations as well as specific antibody titers to RSV, CMV, tetanus, Hib, measles, and 13 serotypes of *S. pneumoniae*.

### Study Subjects

Eligible subjects were male and female aged 2–75 years, inclusive with a confirmed diagnosis of primary immunodeficiency. Prior to enrollment, subjects were required to have received IVIG infusions every 3 or 4 weeks at a dose that had not been changed by >50 % of the mean dose on a mg/kg basis for ≥3 months. Subjects were excluded if they had a history of adverse reactions to blood or blood-derived products, selective IgA deficiency, and history of allergic reaction to products containing IgA or antibodies to IgA, abnormal liver function, deep vein thrombosis, hemolysis, or positive Coombs test or were pregnant or lactating.

### Efficacy Evaluation

The primary efficacy endpoint was the demonstration of a serious bacterial infection (SBI) rate of less than 1.0 per person-year during the 12-month treatment with RI-002. Secondary efficacy endpoints included: the incidence of all infections of any kind, the number of days lost from work/school/usual activity due to infection and their treatment, the number of unscheduled visits to physician/ER due to infections, the time to resolution of infections, the number of hospitalizations and days of hospitalizations due to infections, the number of days of antibiotic therapy (prophylactic and treatment), the relationship among dose, trough level and risk of serious and non-serious infections, and trough total IgG and specific antibody levels.

### Safety Evaluation

Subjects were monitored for adverse events (AEs) and serious adverse events (SAEs) during each infusion and between each visit using subject diaries. AEs were defined as any untoward medical occurrence whether or not the AE was determined to be product related. Subjects regularly completed a subject diary to capture data between study infusions. Subject diaries were used to capture the incidence and duration of infections (SBIs and other infections), use of antibiotics, sick days (missed work or school), unscheduled doctor or hospital visits due to infection, and the incidence of adverse events. These data were reviewed with study personnel at each study visit to confirm their accuracy and completeness. Temporally associated AEs (TAAEs) were adverse events occurring within 72 h of RI-002 infusion. Safety endpoints included the incidence of TAAEs, SAEs, and related SAEs, discontinuation of study treatment, and PIDD related deaths, infusion site reactions, change in vital signs before or after infusion, proportion of infusions with one or TAAEs, and changes in laboratory values.

### Pharmacokinetic Evaluation

The blood samples for PK analysis were obtained beginning after Infusion 7 or Infusion 9 in order to allow for wash out of the previous IG product. Scheduled sampling times were as follows: immediately prior to the infusion, end of the infusion, 60 min, 2 h, 24 h, 48 h, 4 days, 7 days, 14 days, 21 days, and 28 days post-infusion. All PK parameters were calculated using non-compartmental analysis. Only those plasma concentrations equal to or greater than the validated lower limit of quantitation of the individual antibody assays were used in the analysis. Actual sampling times were used in all pharmacokinetic analyses. Per protocol times were used to calculate mean plasma concentrations for graphical displays. Calculated PK parameters included: elimination constant (K), elimination half-life (t½), area under the curve (AUC_(0-t)_) over the 21 or 28-day dosing interval, total plasma clearance (CL), and volume of distribution (Vz). Levels of specific antibody to CMV, tetanus, Hib, measles, and RSV were determined at each time point.

### Statistical Analysis

The Intent-To-Treat (ITT) population included all screened subjects who fulfilled eligibility criteria for RI-002 treatment. All ITT subjects who received at least one RI-002 infusion in the study (referenced to as mITT) were the primary population for efficacy and safety analyses. The PK population includes all mITT subjects who enrolled to participate in the PK portion of the study and who had sufficient plasma samples drawn to derive PK parameters.

Descriptive statistics were provided for the overall population and by treatment regimen (*n*, mean, SD, minimum, median, and maximum for continuous variables and counts and percentages for categorical variables). Infections were identified based on reported treatment emergent adverse events that were mapped to MedDRA ‘Infection and Infestations’ System Organ Class. The concomitant medications records were used to identify all antibiotic medications based on the WHO Drug Coding of ATC level, route and indication. Annualized rate (per person per year) was calculated by total episodes (e.g., total infection events) or total days (e.g., days lost due to infection) divided by total person-years. The one-sided 95 % upper bound for annualized rate was estimated from a general linear model for Poisson distribution with only the intercept parameter. Quartiles and mean days to resolution of infections were estimated using Kaplan-Meier estimates.

The two-sided 90 % confidence intervals were calculated using normal approximation with a continuity correction for proportion of infusions with ≥ 1 TAAEs.

## Results

### Subject Demographics

A total of 75 subjects were screened and 59 subjects, 28 males and 31 females, were enrolled and received at least 1 RI-002 infusion (mITT population). The majority of study subjects were Caucasian (98.3 %), average age was 41.8 years ranging from 3 to 73 years. The study included 48 adult subjects (>16 years of age) and 11 pediatric subjects. The most common PIDD type was common variable immunodeficiency (CVID) in 45 subjects (76.3 % of the subjects). Mean time from diagnosis to study enrollment was 8.66 years (ranged from 0.3 to 32.9 years). Demographic characteristics were similar between the two regimens (Table 1).

**Table 1:** Summary of demographic data for mITT population

	Total (*N* = 59)	3-Week cycle (*N* = 19)	4-Week cycle (*N* = 40)
Age, mean (SE)	41.8 (2.84)	38.6 (4.94)	43.3 (3.48)
Subjects ≤16 years, *n*	11	3	8
Subjects 17 to 64 years, *n*	37	12	25
Subjects ≥65 years, *n*	11	4	7
PIDD diagnosis, *n* (%)			
CVID	46 (77.9)	17 (89.5)	29 (72.5)
X-linked Agammaglobulinemia	6 (10.2)	0	6 (15.0)
Antibody deficiencies	7 (11.9)	2 (10.5)	5 (12.5)
Years since diagnosis, mean (SE)	8.66 (1.1)	6.87 (1.4)	9.51 (1.5)
Median (range)	5.68 (0.3, 32.9)	5.13 (0.5, 21.7)	5.82 (0.3, 32.9)

### Efficacy

RI-002 was efficacious in the treatment of subjects with PIDD aged 3 to 74 years, over a 1-year period. The efficacy of RI-002 (Table 2) was demonstrated by the absence of any SBIs in the study population. The observed incidence of zero (0) SBIs in 55.88 subject years (20,396 total study days) of treatment resulted in a SBI rate below the criteria of <1.0 SBI per subject per year set by FDA. Other key efficacy secondary endpoints included lost days from work/school/daycare due to infection (1.66 days per subject per year), unscheduled emergency room/medical visits due to infection (0.966 visits per subject per year), and hospitalizations due to infection (0.018 hospitalizations per subject per year).

**Table 2:** Summary of Annualized rates for infections, antibiotics use, days lost for work/school/daycare, unscheduled visits, and hospitalizations

	Total	3-Week cycle	4-Week cycle
Number of mITT Subjects	59	19	40
Total number subject-years	55.9	17.3	38.6
Infections			
Number of Serious acute bacterial infections (SBIs)	0	0	0
Rate of SBIs per person per years (one-sided 99 % upper bound)	0.000 (<1.0)	0.000 (<1.0)	0.000 (<1.0)
Total infections of any kind/seriousness	192	62	130
Infections per subject per year (one-sided 95 % upper bound)	3.436 (3.869)	3.584 (4.417)	3.370 (3.893)
Antibiotic use for therapy			
Number of subjects, *n* (%)	37 (62.7)	12 (63.2)	25 (62.5)
Days per subject per year	32.9	41.2	29.2
Days off school/work/day care due to infection			
Number of Subjects, *n* (%)	23 (39.0)	7 (36.8)	16 (40.0)
Total days	93	27	66
Days per subject per year (one-sided 95 % upper bound)	1.66 (1.97)	1.56 (2.14)	1.71 (2.09)
Unscheduled visits to the ER and physician due to infection			
Number of subjects, *n* (%)	24 (40.7)	9 (47.4)	15 (37.5)
Total days	54	18	36
Days per subject per year (one-sided 95 % upper bound)	0.97 (1.21)	1.04 (1.53)	0.93 (1.23)
Hospitalization due to infection			
Number of subjects, *n* (%)	1 (1.7)	0	1 (2.5)
Number of days	5	0	5
Hospitalizations per subject per year	0.018	0	0.026

Analysis of trough concentrations of IgG and specific antibodies demonstrated that administration of RI-002 at doses of 284–1008 mg/kg every 3 or 4 weeks generally maintained concentrations similar to those present at baseline. Trough RSV titers, however, were an exception to this observation with levels increasing after the first RI-002 infusion and remaining elevated throughout the study. Increase in antibody levels to all pathogens tested were observed in subjects who received RI-002 and those subjects who received doses of ≥500 mg/kg demonstrated even higher specific antibody levels to all pathogens compared to those receiving doses ≤ 500 mg/kg.

Although this study was not powered to identify differences in safety or efficacy between 3 and 4-week cycles, subjects dosed with RI-002 on a 3-week cycle were observed to have higher trough IgG concentrations compared to subjects on a 4-week cycle but there was no evidence suggesting a difference in efficacy or safety outcomes based on the treatment cycle. Both treatment cycles were successful in maintaining trough IgG concentrations well above the targeted threshold of 500 mg/dL, with mean IgG trough levels ranging from 1051.6 to 1194.9 mg/dL for subjects on a 3-week cycle, and 913.3 to 978.5 mg/dL for subjects on a 4-week cycle.

### Pharmacokinetics

**Fig. 1:**
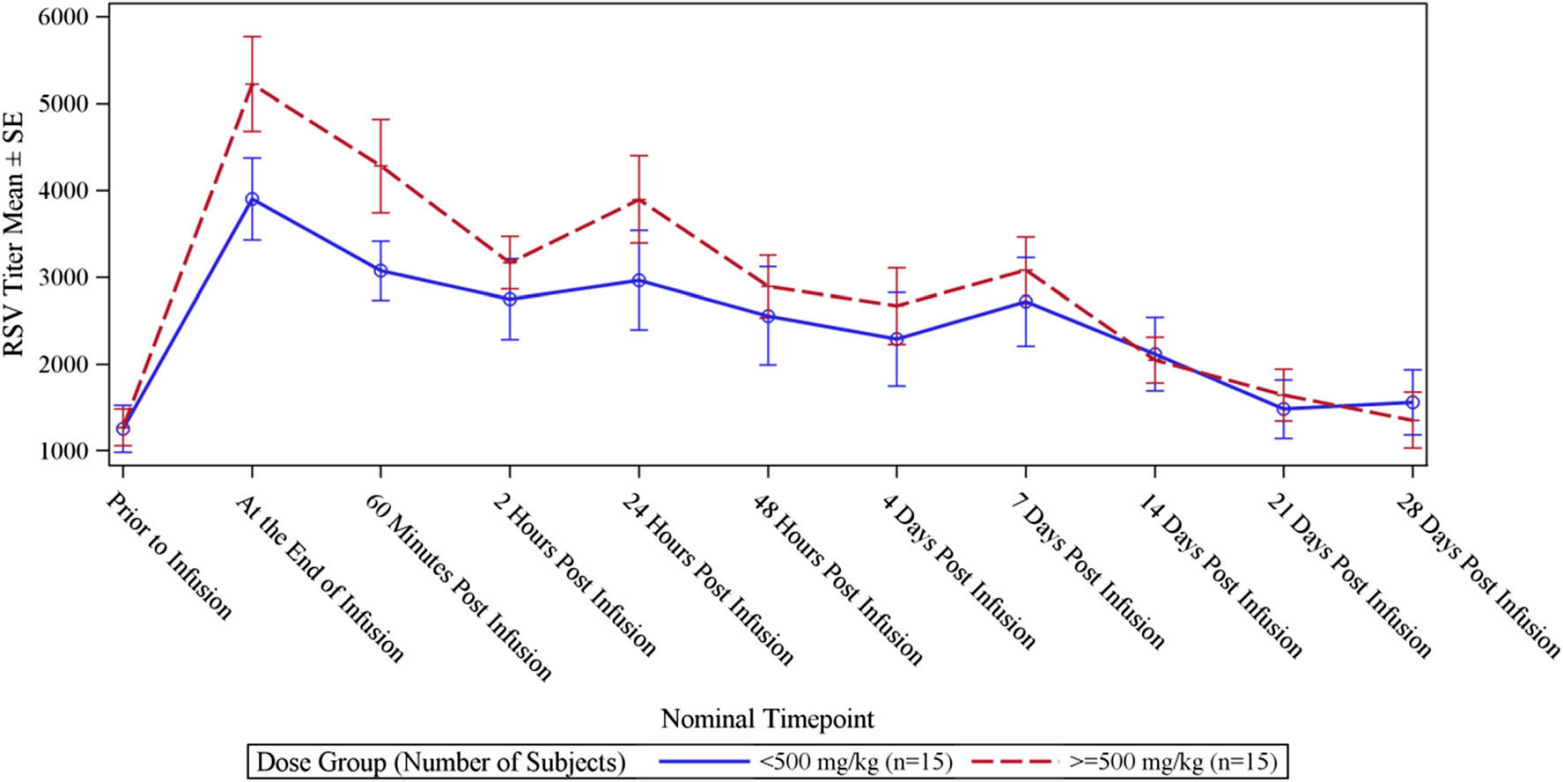
Neutralizing antibodies to RSV before and post-infusion by dose and nominal time point post infusion – PK evaluable set (*N* = 30)

Thirty subjects, 20 subjects on a 4-week infusion cycle and 10 subjects on a 3-week infusion cycle, participated in the PK portion of the study. Administration of RI-002 with either regimen resulted in increases in IgG and in CMV, tetanus, Hib, measles, RSV, and *S. pneumoniae* serotype antibodies with the greatest increase in C_max_ observed in antibodies to RSV. There was a 5.47-fold increase in the titer of anti-RSV neutralizing antibody immediately after infusion of the IVIG and a 6.79-fold increase in those subjects who received greater than 500 mg/kg (Table 3). In this calculation, baseline was that value of anti-RSV measured immediately prior to entry into the trial. Figure 1 shows the pharmacokinetics of anti-RSV antibodies, and in this situation, baseline was the value of anti-RSV that was measured in the subjects enrolled in the PK portion of the study immediately prior to infusion of the seventh or ninth dose of IVIG. RI-002 is manufactured to contain standardized, elevated levels of neutralizing antibodies to RSV, and all lots are tested for release potency for RSV antibody levels. There was no apparent difference between groups in the mean plasma concentrations of IgG and the mean values for C_max_ despite a slightly larger AUC over the dosing interval due to the 7-day longer period. Although the mean values for t½ should be considered as qualitative because of the period of time over which λz was measured, they are consistent with literature values for the t½ of IgG.

**Table 3:** Maximum observed neutralizing antibodies to RSV (Cmax) post-infusion and fold change from baseline – PK evaluable set (*N* = 30)

Dose group	Statistics	Baseline	Cmax	Fold change (Cmax/baseline)
All doses	*n*	29	30	29
	Mean (SE)	1260.00 (272.885)	4770.20 (392.025)	5.47 (0.534)
	95 % CI	701.02, 1818.98	3968.42, 5571.98	4.37, 6.56
Dose < 500 mg/kg	*n*	15	15	15
	Mean (SE)	1532.27 (499.761)	4145.93 (529.355)	4.23 (0.596)
	95 % CI	460.39, 2604.15	3010.58, 5281.29	2.95, 5.51
Dose≥500 mg/kg	*n*	14	15	14
	Mean (SE)	968.29 (177.181)	5394.47 (548.453)	6.79 (0.776)
	95 % CI	585.51, 1351.06	4218.15, 6570.78	5.11, 8.47

Although there was a range of RI-002 doses within each group, there was no apparent relationship between AUC over the dosing interval and either total dose or weight adjusted dose for either cohort (Table 4). Taken as a whole, the increases in the RSV, CMV, tetanus, Hib, measles, and *S. pneumoniae* antibodies were not affected by the treatment intervals used in this study. The mean values for all antibodies measured at baseline were comparable to the pre-infusion titers for both cohorts, and the maximum titers were higher than the baseline and pre-infusion titers. There did not appear to be a difference in exposure to IgG as a function of sex or age group. Analysis of baseline corrected plasma IgG concentrations resulted in comparisons between the 3 and 4-week cycle cohorts that were comparable to those using plasma concentrations without baseline correction.

**Table 4:** Total IgG pharmacokinetic parameter estimates (PK population)

Statistic	3-Week cycle (*N* = 10)		4-Week cycle (*N* = 20)	
	Mean ± SD (*n*)	CV%	Mean ± SD (*n*)	CV%
C_max_ (mg/dL)	2427 ± 452 (10)	18.63	2227 ± 584 (20)	26.21
C_min_ (mg/dL)	1152 ± 308 (10)	26.73	954 ± 245 (20)	25.65
T_max_ (h)^a^	2.93 [1.80,4.52] (10)	NA	2.78 [1.43,99.08] (20)	NA
AUC_tau_ (day mg/dL)	32,128 ± 7020 (10)	21.85	35,905 ± 9351 (20)	26.04
t½ (day)	28.47 ± 4.38 (6)	15.38	39.70 ± 11.57 (13)	29.13
CL (mL/kg/day)	1.68 ± 0.43 (10)	25.42	1.47 ± 0.50 (20)	33.63
V_ss_ (dL/kg)	76.79 ± 13.45 (6)	17.52	89.57 ± 26.16 (13)	29.21

*AUC*_tau_ steady-state area under the plasma concentration versus time curve with tau=dosing interval, *CL* total body clearance, *C*_ma*x*_ maximum concentration, *C*_min_ minimum concentration, *CV* coefficient of variation, *n* number of subjects, *NA* not applicable, *SD* standard deviation, *T*_max_ time of maximum concentration, *t½* terminal half-life, *V*_ss_ volume of distribution steady-state

^a^ Units median [range] (*n*)

### Safety

RI-002 was well tolerated in this study. The proportion of study infusions with a TAAE was 0.142 with an upper one-sided 95 % confidence limit of 0.164, indicating that the study met the target (<0.40) for this safety endpoint. During the study, 43 (72.9 %) subjects experienced at least one TAAE, and TAAEs were experienced during 113 (14.2 %) study infusions (Table 5).

**Table 5:** Summary of temporally associated AEs (TAAEs)

	Total	3-Week cycle	4-Week cycle
Total number of subjects	59	19	40
Total number of infusions	793	294	499
Number of infusions with ≥ 1 TAAEs	113	36	77
Total number of TAAEs	158	47	111
Mean number of TAAEs per infusion	0.199	0.160	0.222
Proportion of infusions with ≥ 1 TAAEs (one-sided 95 % upper limit)	0.142 (0.164)	0.122 (0.156)	0.154 (0.182)
Subjects with ≥ 1 TAAE, *n* (%)			
Within 1 h	28 (47.5)	7 (36.8)	21 (52.5)
Within 24 h	40 (67.8)	13 (68.4)	27 (67.5)
Within 72 h	43 (72.9)	15 (78.9)	28 (70.0)
Subjects with ≥ 1 study drug-related TAAE, *n* (%)			
Within 1 h	14 (23.7)	5 (26.3)	9 (22.5)
Within 24 h	21 (35.6)	7 (36.8)	14 (35.0)
Within 72 h	21 (35.6)	7 (36.8)	14 (35.0)
TAAEs reported by ≥ 5 % total subjects, *n* (%)			
Headache	14 (23.7)	3 (15.8)	11 (27.5)
Sinusitis	6 (10.2)	2 (10.5)	4 (10.0)
Nausea	5 (8.5)	1 (5.3)	4 (10.0)
Acute sinusitis	4 (6.8)	1 (5.3)	3 (7.5)
Fatigue	4 (6.8)	2 (10.5)	2 (5.0)
Muscle spasms	4 (6.8)	1 (5.3)	3 (7.5)
Adverse drug reaction	3 (5.1)	0	3 (7.5)
Bronchitis	3 (5.1)	0	3 (7.5)
Diarrhea	3 (5.1)	1 (5.3)	2 (5.0)
Epistaxis	3 (5.1)	0	3 (7.5)
Myalgia	3 (5.1)	2 (10.5)	1 (2.5)
Oropharyngeal pain	3 (5.1)	0	3 (7.5)
Pain in extremity	3 (5.1)	0	3 (7.5)
Pruritus	3 (5.1)	0	3 (7.5)

There were 793 infusions administered during the study, with an average of 13.4 infusions administered per subject. Of 793 study infusions, one was not completed as planned, and two infusions (in 2 different subjects) were interrupted due to an AE and completed on the next day. The mean (SE) planned weight adjusted dose of RI-002 administered during this study was 505.2 (4.84) mg/kg, with a range of 284 to 1008 mg/kg.

The majority of subjects in this study did not experience any infusion-related AEs or complications. Study infusions were generally well tolerated with 95.8 % of infusions administered at the maximum allowed infusion rate of 8 mg/kg/min RI-002 infusion rates ranged from 0.5 to 13.3 mg/kg/min during the study. The use of pre-medication was limited to seven study infusions (out of a total of 793 total infusions) in seven subjects (0.009 %).

No serious bacterial infections occurred during the study. A total of 618 treatment emergent AEs (TEAEs) were experienced by 58 subjects (98.3 %) during the course of this study (Table 6). The most frequently reported TEAEs, regardless of relationship to study drug, included: headache, sinusitis, diarrhea, viral gastroenteritis, nasopharyngitis, and upper respiratory tract infection. A total of 55 TEAEs were determined to be study drug related in 26 (44.1 %) study subjects. The most frequently reported study drug-related TEAE was headache.

**Table 6:** Summary of treatment emergent adverse events

Category	Total (*N* = 59)		3-Week cycle (*N* = 19)		4-Week Cycle (*N* = 40)	
	No.	*n* (%)	No.	*n* (%)	No.	*n* (%)
Subjects with ≥ 1 TEAE	618	58 (98.3)	180	18 (94.7)	438	40 (100)
Subjects with ≥ 1 Mild TEAE	411	55 (93.2)	119	18 (94.7)	292	37 (92.5)
Subjects with ≥ 1 Moderate TEAE	186	48 (81.4)	59	17 (89.5)	127	31 (77.5)
Subjects with ≥ 1 Severe TEAE	21	9 (15.3)	2	2 (10.5)	19	7 (17.5)
Subjects with ≥ 1 study drug related TEAE	55	26 (44.1)	22	10 (52.6)	33	16 (40)
Subjects with ≥ 1 related and severe TEAE	3	2 (3.4)	1	1 (5.3)	2	1 (2.5)
Subjects with ≥ 1 SAE	2	2 (3.4)	0	0	2	2 (5.0)
Subjects withdrawals due to an AE	2	2 (3.4)	0	0	2	2 (5.0)
Deaths	0	0	0	0	0	0
TEAE occurred ≥ 10 % total subjects						
Headache	42	22 (37.3)	13	6 (31.6)	29	16 (40.0)
Sinusitis	31	16 (27.1)	11	5 (26.3)	20	11 (27.5)
Diarrhea	16	14 (23.7)	3	3 (15.8)	13	11 (27.5)
Gastroenteritis viral	16	13 (22.0)	5	4 (21.1)	11	9 (22.5)
Nasopharyngitis	23	13 (22.0)	11	6 (31.6)	12	7 (17.5)
Upper respiratory tract infection	15	13 (22.0)	5	4 (21.1)	10	9 (22.5)
Bronchitis	14	12 (20.3)	1	1 (5.3)	13	11 (27.5)
Nausea	17	12 (20.3)	2	2 (10.5)	15	10 (25.0)
Study drug-related TEAEs occurring in > 20 % of total subjects						
Headache	10	8 (13.6)	3	3 (15.8)	7	5 (12.5)

No. = total number of events

There were no SAEs reported during the trial attributable to the study drug. Two serious adverse events (SAEs) were reported, one instance each of post-operative wound infection and migraine. Neither SAE was associated with the study drug; each event was experienced as a single occurrence in a single subject.

There was a low incidence of adverse infusion reactions, with a total of 31 events experienced by 18 subjects (30.5 %) during 29 infusions (3.7 %). The most frequently reported events included headache and myalgia. A total of four infusion site reactions were reported in this study in two subjects. The infusion site reactions reported included infusion site extravasation and infusion site pain. There was no evidence of a trend towards significant changes in any of the laboratory assessments during treatment with study medication. End of study laboratory assessments were generally similar to screening assessments, with no trends noted. There was no laboratory evidence of any instances of a thrombotic event, acute renal failure, hemolysis event, viral transmission, aseptic meningitis, or transfusion-related acute lung injury.

## Discussion

Among the viral infections that cause substantial morbidity, respiratory syncytial virus is a particular problem, especially in patients with coexistent pulmonary disease [19, 20]. In 1996, an anti-RSV hyper-immune globulin product, RSV-IVIG (RespiGam®), was approved for the prevention of severe respiratory syncytial virus infection in premature infants whose gestational age was less than 32 weeks. Studies suggested that RSV-IVIG reduced disease severity in infants who became infected with RSV, shortening hospital stays compared to age-matched controls [15, 16, 21]. Despite these beneficial effects, RSV-IVIG was voluntarily removed from the market by the manufacturer in favor of an intramuscularly administrated monoclonal anti-RSV antibody palivizumab (Synagis®). There are no direct head to head clinical studies comparing the efficacy of RSV-IVIG to palivizumab [17]. While both appear comparable in their ability to prevent RSV infections in low birth weight infants, there appear to be certain clinical advantages of the polyclonal antibody [17]. In the PREVENT study, not only was RSV-IVIG able to prevent RSV infections but it also reduced the incidence of other, non-RSV-related upper respiratory diseases [15]. Another study demonstrated that high-risk infants receiving RSV-IVIG had a statistically significant reduction in the overall frequency of otitis media compared with placebo-treated subjects, also suggesting that polyclonal antibody activity to other pathogens may have contributed to this reduction observed in the incidence of otitis media [21]. Interestingly, in the current study, there was only one single reported case of otitis media, an exceedingly low incidence for this population of subjects in the study. Recently published studies in the cotton rat demonstrated that RI-002 can not only reduce the viral load in normal and immune suppressed cotton rats that have been infected with RSV, but that it can dramatically reverse the intense inflammatory changes that are observed in their pulmonary tissue [22].

Because of the historical data suggesting the potential value of a polyclonal anti-RSV, IG product for the immune-compromised patient population, a new Ig formulation was developed. The new product, RI-002, is an IVIG that is manufactured and standardized to contain elevated levels of anti-RSV neutralizing antibody and, in this regard, is comparable to RespiGam® [22–24]. However, this new product has the added advantage that it is manufactured to comply with the FDA guidance for IG products for the treatment of patients with PIDD [18]. Similar to other IGs, this IVIG (RI-002) was made from plasma collected from more than 1000 plasma donors and was manufactured using an FDA-approved fractionation and purification process [25, 26]. Unlike other IGs, the plasma that was used to produce RI-002 was collected from donors who were identified and selected to have high neutralizing antibody titers to RSV. The selection was based on testing results obtained utilizing a validated RSV live-virus microneutralization assay. There was as great as a 6.79-fold increase in the levels of anti-RSV neutralizing antibody that was measured in subjects who were on the dosing regimen of greater than 500 mg/kg. Interestingly, the lots of IVIG produced from RSV hyperimmune donors were also found to have significantly elevated antibody titers to other respiratory viruses (parainfluenza type 1 and 3, influenza, coronavirus, and metapneumovirus) as compared to ten other commercial lots of IG [27]. This preparation of RI-002, therefore, can be regarded as having a unique antibody profile for multiple respiratory viruses that differentiates it from other commercially available IGs. Viewed in the context of data from other IG trials, there are many of the secondary efficacy endpoints, for example number of days missed from work/school/daycare (1.66 per subject per year), hospitalization per subject per year (0.018), and unscheduled visits to the physician or emergency room (0.72 per subject per year) compared favorably to these published trials [25, 28–30]. This study demonstrated excellent outcomes that might reflect the unique antibody profile of RI-002.

The efficacy of RI-002 is likely the result of the maintenance of stable IgG trough levels as well as specific antibody levels throughout the study conferring the expected protective effects of IG therapy. The pharmacokinetic properties of RI-002 were comparable to previous reports of IVIG preparations. All subjects maintained average trough levels of serum Ig of approximately 1000 mg/kg. There were significant post-infusion increases in all of the specific antibody titers measured with a greater than 6-fold increase seen in the concentration of RSV-neutralizing antibody immediately post-infusion in patients receiving greater than 500 mg/kg of IG.

There were no acute serious bacterial infections reported. The single infection that required hospitalization was believed to be secondary to a cat scratch that infected a recovering, post-operative, shoulder joint repair wound. That subject was non-compliant and missed his regularly scheduled dose of RI-002.

RI-002 was safe and well tolerated. The rates of temporally associated and treatment associated adverse events were well within the FDA guidance and comparable to the rates of adverse events that have occurred in studies of other IVIG products. Infusions were completed without interruption, discontinuation or rate reduction in 790 of 793 (99.6 %) infusions.

Two subjects each reported one SAE (post-operative wound infection and migraine) during this study, and neither was associated with study drug. The subject with the post-operative wound infection SAE discontinued from the study whereas the subject with SAE of migraine completed the study. One subject had an adverse drug reaction and discontinued from the study. Three other discontinuations were due to other causes (one subject became pregnant, one subject was relocated, and 1 was discontinued due to sponsor because of subject non-compliance).

## Conclusions

RI-002 is a safe, well-tolerated, highly effective immunoglobulin replacement for PIDD, manufactured with a unique antibody profile from a donor population tested to have elevated anti-RSV-neutralizing antibody titers. The unexpectedly low rate of infection-related outcomes suggests that the unique, anti-viral antibody profile of RI-002 may be important in the prevention of infections in PIDD subjects and raises novel questions about the role of viral infections in the pathogenesis of the infectious complications of PIDD. Since it has been reported that pulmonary viral infections are often the antecedent to bacterial pulmonary infections, it is tempting to speculate that an IVIG that has high titer antibodies to viruses may also have an impact on reducing the incidence of bacterial infections.

## References

[1] Boyle ML, Scalchunes C (2008). Impact of intravenous immunoglobulin (IVIG) treatment among patients with primary immunodeficiency diseases. Pharm Policy Law.

[2] Busse PJ, Razvi S, Cunningham-Rundles C (2002). Efficacy of intravenous immunoglobulin in the prevention of pneumonia in patients with common variable immunodeficiency. J Allergy Clin Immunol.

[3] Cunningham-Rundles C, Maglione PJ (2012). Common variable immunodeficiency. J Allergy Clin Immunol.

[4] Orange JS, Hossny EM, Weiler CR (2006). Use of intravenous immunoglobulin in human disease: a review of evidence by members of the Primary Immunodeficiency Committee of the American Academy of Allergy, Asthma and immunology. J Allergy Clin Immunol.

[5] Albin S, Cunningham-Rundles C (2014). An update on the use of immunoglobulin for the treatment of immunodeficiency disorders. Immunotherapy.

[6] Quinti I (2011). Effectiveness of immunoglobulin replacement therapy on clinical outcome in patients with primary antibody deficiencies: results from a multicenter prospective cohort study. J Clin Immunol.

[7] Orange JS, Grossman WJ, Navickis RJ (2010). Impact of trough IgG on pneumonia incidence in primary immunodeficiency: a meta-analysis of clinical studies. Clin Immunol.

[8] Lucas M, Lee M, Lortan J (2010). Infection outcomes in patients with common variable immunodeficiency disorders: relationship to immunoglobulin therapy over 22 years. J Allergy Clin Immunol.

[9] Berger M (1999). Goals of therapy in antibody deficiency syndromes. J Allergy Clin Immunol.

[10] Bonagura VR, Marchlewski R, Cox A (2008). Biologic IgG level in primary immunodeficiency disease: the IgG level that protects against recurrent infection. J Allergy Clin Immunol.

[11] Furst DE (2009). Serum immunoglobulins and risk of infection: how low can you go?. Semin Arthritis Rheum.

[12] Quartier P, Debré M, De Blic J (1999). Early and prolonged intravenous immunoglobulin replacement therapy in childhood agammaglobulinemia: a retrospective survey of 31 patients. J Pediatr.

[13] Sastre P, Melero JA, Garcia-Barreno B (2004). Comparison of antibodies directed against human respiratory syncytial virus antigens present in two commercial preparations of human immunoglobulins with different neutralizing activities. Vaccine.

[14] Groothuis JR, Hoopes JM, Hemming VG (2011). Prevention of serious respiratory syncytial virus-related illness II: immunoprophylaxis. Adv Ther.

[15] The PREVENT Study Group. Reduction of respiratory syncytial virus hospitalization among premature infants and infants with bronchopulmonary dysplasia using respiratory syncytial virus immune globulin prophylaxis. Pediatrics. 1997;99(1):93–9. 10.1542/peds.99.1.93 8989345

[16] Groothuis J (1993). Prophylactic administration of respiratory syncytial virus immune globulin to high risk infants and young children. NEJM.

[17] American Academy of Pediatrics: Committee on Infectious Diseases and Committee on Fetus and Newborn (1998). Prevention of respiratory syncytial virus infections: indications for the use of palivizumab and update on the use of RSV-IGIV. Pediatrics.

[18] FDA Guidance. Guidance for industry safety, efficacy, and pharmacokinetic studies to support marketing of immune globulin intravenous (human) as replacement therapy for primary humoral immunodeficiency. June 2008.

[19] Paes B, Manzoni P (2011). Special populations: do we need evidence from randomized controlled trials to support the need for respiratory syncytial virus prophylaxis?. Early Hum Dev.

[20] Faroux B (2009). Insights from the Sixth Global Experts’ Meeting (GEM) on respiratory viruses: special populations. Paediatr Respir Rev.

[21] Simoes EA, Groothius JR, Tristram DA (1996). Respiratory syncytial virus-enriched globulin for the prevention of acute otitis media in high-risk children. J Pediatr.

[22] Boukhvalova MS (2016). Treatment with novel RSV Ig RI-002 controls viral replication and reduces pulmonary damage in immunocompromised Sigmodon hispidus. Bone Marrow Transplant.

[23] Falsey AR. Polyclonal human intravenous immune globulin (IGIV) with high-levels of RSV neutralizing antibodies: a summary of animal and human studies. Annual Conference of the Canadian Blood and Marrow Transplant Group June 11–14, 2014.

[24] Mond J. Treatment of normal and immune suppressed cotton rats with IVIG containing high neutralizing titer anti-RSV antibody. 9th International Respiratory Syncytial Virus Symposium November 2014.

[25] Wasserman RL (2014). A new intravenous immunoglobulin: bivigam for primary humoral immunodeficiency. Expert Rev Clin Immunol.

[26] BIVIGAM Prescribing Information. 2013.

[27] Orange JS, Du W, Falsey AR (2015). Therapeutic immunoglobulin selected for high antibody titer to RSV also contains high antibody titers to other respiratory viruses. Front Immunol.

[28] Berger M, Pinciaro PJ, Althaus A (2010). Efficacy, pharmacokinetics, safety, and tolerability of Flebogamma 10% DIF, a high-purity human intravenous immunoglobulin, in primary immunodeficiency. J Clin Immunol.

[29] Jolles S, Bernatowska E, de Garcia J (2011). Efficacy and safety of Hizentra® in patients with primary immunodeficiency after a dose-equivalent switch from intravenous or subcutaneous replacement therapy. Clin Immunol.

[30] Stein MR, Nelson RP, Church JA (2009). Safety and efficacy of Privigen, a novel 10% liquid immunoglobulin preparation for intravenous use, in patients with primary immunodeficiencies. J Clin Immunol.

